# Machine Learning Approaches for the Frailty Screening: A Narrative Review

**DOI:** 10.3390/ijerph19148825

**Published:** 2022-07-20

**Authors:** Eduarda Oliosi, Federico Guede-Fernández, Ana Londral

**Affiliations:** 1 Value for Health CoLAB, 1150-190 Lisboa, Portugal; mariaeduardaoliosi@icloud.com (E.O.); federico.guede@vohcolab.org (F.G.-F.); 2 LIBPhys (Laboratory for Instrumentation, Biomedical Engineering and Radiation Physics), NOVA School of Science and Technology, NOVA University of Lisbon, 2829-516 Caparica, Portugal; 3 Comprehensive Health Research Center, NOVA Medical School, NOVA University of Lisbon, 1150-082 Lisboa, Portugal

**Keywords:** frailty, indicators, screening, artificial intelligence, healthcare

## Abstract

Frailty characterizes a state of impairments that increases the risk of adverse health outcomes such as physical limitation, lower quality of life, and premature death. Frailty prevention, early screening, and management of potential existing conditions are essential and impact the elderly population positively and on society. Advanced machine learning (ML) processing methods are one of healthcare’s fastest developing scientific and technical areas. Although research studies are being conducted in a controlled environment, their translation into the real world (clinical setting, which is often dynamic) is challenging. This paper presents a narrative review of the procedures for the frailty screening applied to the innovative tools, focusing on indicators and ML approaches. It results in six selected studies. Support vector machine was the most often used ML method. These methods apparently can identify several risk factors to predict pre-frail or frailty. Even so, there are some limitations (e.g., quality data), but they have enormous potential to detect frailty early.

## 1. Introduction

With the growing aging population worldwide, an important subject matter is “frailty” (or fragility) which is closely age-related [1]. Living longer can lead to a longer period of frailty with increased demand for care [2]. The proportion of the elderly is expected to be approximately 30% of the population by 2060, in Europe [3]. Moreover, aging expenditures are projected to increase by 1.5 percentage points of GDP, from 26.8% in 2013 to 28.3%, in 2060 [4]. Frailty is a broad term used to denote a complex clinical condition [5,6] that can be defined as a medical syndrome caused by multisystem dysregulation and contributors. In addition, it is characterized by loss of health reserves (e.g., physical fitness), reduced physiologic function, and impaired homeostasis, which increases an individual’s vulnerability, resulting in risk for early dependency, morbidity, and/or death when exposed to stressors. In summary, the frailty syndrome involves the main domains: physical, psychological, social, cognitive, and environmental [6,7,8,9,10,11]. Moreover, frailty characterizes a state of impairments that increase the risk of negative health outcomes such as physical limitation, falls, fractures, disability, morbidity, dependence, hospitalization, institutionalization, lower quality of life, and premature death [4,12,13,14,15].

Barriers to implementing frailty screening in clinical settings still exist as a lack of consensus on the assessment tool best suited to each domain and undetermined cost-effectiveness [16]. Moreover, it is imperative to note that the frailty assessment tools can provide different data regarding the incidence of frailty [17]. The identification of frailty might seem an ideal way to identify the elderly who need additional healthcare support services. In a recent review, Liotta and colleagues (2018), from a public health perspective, stressed that it is vital to identify factors that contribute to successful health and social care interventions and to the health systems’ sustainability [18]. Nevertheless, there is a lack of substantial research evidence to support this strategy and to identify the most effective tools to detect frailty [10]. In addition, there is no consensus about the key components and assessment of frailty [19].

In a systematic review, Sutton and colleagues (2016) identified 38 multi-component frailty assessment tools where, surprisingly, only 5% (2/38) of the frailty assessment tools had evidence of reliability and validity that was within statistically significant parameters and of fair–excellent methodological quality: the Frailty Index—Comprehensive Geriatric Assessment and the Tilburg Frailty Indicator [20]. In addition, a score or set of criteria was used, developed, and validated to identify frailty. The most common frailty instruments used in research and clinical practice are the Fried frailty phenotype (FP), which is based on five items (slow walking speed, weak grip strength, low physical activity, unintended weight loss, and exhaustion), minimum of three of five criteria for classifying as frailty [21,22,23]. Nevertheless, there is insufficient evidence to determine the best tool for use in research and clinical practice [20].

According to an umbrella review, despite these broadly used conventional methods, few frailty measures seem to be valid, reliable, diagnostically accurate, and virtuous predictive abilities. Moreover, they reported that the Frailty Index (and gait speed) emerged as the most useful in routine care and community settings [24]. The traditional measurements of frailty have potential limitations and challenges: for example, single measures of physical performance (such as timed-get-up-and-go) or a set of physical features (such as FP) are clinically suitable and validated to predict poor outcomes in older adults. Nevertheless, they have shown low consistency, accuracy, reliability, and inter-rater understanding. Moreover, these measures require specialized equipment (e.g., dynamometer to grip strength), not always clinically viable (e.g., for patients with dementia), and also require a manual evaluation process (e.g., timed-get-up-and-go) that is subject to operator error due to the need for training beyond time to administer [25]. Furthermore, the prevalence of frailty varies across settings and adopted tests, making it difficult to scale to the population level [21,22,25,26]. In this view, an alternative is exploring approaches to screening frailty from routinely collected data (e.g., medical claims, prescriptions, administrative data, and individual records) [25].

The presented work is part of the Frailcare.AI project. Its primary objective is to develop intelligent tools that aim to improve pathways for the identification of fragility in senior citizens in the Portuguese population. This paper aims to review tools and clinical indicators for identifying early frailty and supply evidence for developing innovative tools and artificial intelligence (AI) technologies to support frailty care. This review provides recent evidence for the assessment and screening of frailty. It reviews the existing tools and clinical indicators for complex frailty, focusing on measures extracted from healthcare datasets. We seek to improve knowledge and application opportunities for machine learning (ML). This intelligent screening tool relies on an approach that includes ML methods.

### 1.1. Background

ML methods can adapt conventional frailty screening methods validated in previous studies. While AI is a subfield of computer science dedicated to providing computers with intelligent problem-solving capabilities, including planning, reasoning, perception, or learning (i.e., AI aims to mimic human intelligence and behavior through systems), ML, a subfield of AI, provides algorithms that build mathematical models based on sampled data. These models map input data to desired outputs. Inputs can be images and an arbitrary sequence of numerical or categorical data. The inputs are also known as features [27,28,29,30].

The AI resource includes advanced algorithms and methods that do not even process quantitative data. Consequently, comparing ML to traditional statistical methods makes it coherent. Conventional statistical models focus on discovering interactions and confidence intervals between data points and outcomes; comparatively, ML approaches seek to reach high prediction accuracy, placing less emphasis on whether it is possible to interpret the model. Prediction is critical in ML to generate otherwise unavailable data. Moreover, ML is often better fitting for significant input variables (e.g., time series from biosignals), and the traditional analysis with statistical models is intended for data with tens of input columns [28].

#### 1.1.1. Decision Trees

Decision tree (DT) classification is broadly used for different classification tasks (for example, pattern recognition). DTs make their decisions from the root, all the way up to the branches. The DT approach essentially partitions the space into subspaces by computing the decision boundaries for each node, and it continues adding inputs to the tree nodes until no further improvement can be made to the prediction results. The leaf nodes in the decision tree are labeled according to the groups in the classification problem [31,32].

#### 1.1.2. K-Nearest Neighbours

K-nearest neighbor (KNN) is among the generally used classification approaches. Its algorithm does not create any model through learning strategies. Its training is based on sorting the class labels of the training dataset together with the feature vectors for each record. The accuracy of this model is comparable to more complicated classifiers [31,33].

#### 1.1.3. Support Vector Machine

Support vector machine (SVM) is a supervised classification algorithm; in supervised learning, the models are trained based on given examples, containing inputs and desired outputs provided by an expert (e.g., physical therapist). The SVM has been applied to many real-world classification problems because of its effectiveness, such as pattern recognition for text classification and bioinformatics systems. SVMs are robust to overfitting and have a prominent generalization capability, as well as being good at handling complex, nonlinear scenarios and tending not to overfit. Moreover, SVM is robust to bias and variance of data and results in accurate predictions for either binary or multiclass classifications. As such, SVM has been broadly used in health research, for example, to identify imaging biomarkers of neurological and psychiatric disease, cancer diagnosis, and early detection of Alzheimer’s, among others [29,31,32,33,34].

#### 1.1.4. Artificial Neural Networks

As a brain’s neurons, the artificial neural networks (ANN or NN) are a class of nonlinear statistical algorithms modeled, able to process information. Thus, this approach is defined by how the components of the network are linked and the weights of these connections. This learning process constructs derived parameters as linear combinations of the input parameters and then further models the outcome as a nonlinear constructions of these derived parameters. Although they are excellent at handling many inputs, they are rather computationally costly [32].

#### 1.1.5. Random Forest

The random forest (RF) consists of many decision trees that operate as an ensemble. Each tree provides a class prediction, and the prediction with the most votes turns into the overall model prediction. Therefore, this method is a random forest consisting of a set of individual decision trees; hence, individual errors of the trees are decreased. RF results in a good performance on imbalanced datasets while handling missing values well. These models are not substantially affected by outliers in data. Such decision trees are designed to have a low correlation to each other to encourage range among the trees. Moreover, RFs use the rules of bootstrapping and aggregating to build trees based on several subsets of the training data using different subsets of features [21,35].

#### 1.1.6. Extreme Gradient Boosting

Extreme gradient boosting (XGBoost) is a supervised machine learning model. This method builds a robust model created on weaker models that are short decision trees. The XGBoost works on building a new weak model designed to predict the residual values between the ground truth and the robust model. These weak models are then added to the overall robust model. The predictions of the models are added simultaneously to make the final prediction. The main benefits are execution speed and model performance. These models use boosting, an ensemble method where each tree or model corrects errors made by earlier trees. XGBoost requires minimal feature engineering, allowing steps such as normalizations and scaling to be omitted, and outliers have little impact [21,35,36].

## 2. Methods

### Search Strategy and Data Extraction

Studies were sought using general (Web of Science and Google Scholar) and healthcare (PubMed and The Lancet) databases. Two independent reviewers reviewed all the titles and abstracts in the first selection step. Three keywords were used without period restriction: Frailty screening, as this was the focus of this review, artificial intelligence (AI), and machine learning because ML was considered a subarea of AI. The study inclusion criteria were (i) it described frailty screening tools; (ii) the population was presented with pre-frail or frail conditions/concepts; (iii) studies about frailty indicators, validity studies, articles on frailty screening (frailty assessment, detection, or prediction), and contained significant determinants of frailty; (iv) or if they had a combination of all these criteria. The exclusion criteria were (i) frailty studies about intervention or prevalence; (ii) frailty screening through the inertial sensors; (iii) non-peer-reviewed and academic studies; (iv) all types of reviews (e.g., umbrella and systematic) or case reports or non-English language. There is no existing restriction to frailty screening assessment tools.

## 3. Results

The selection process produced six studies relevant to the aim of this review. Table 1 provides an overview of the selected studies in the frailty screening for ML methods. In general, all studies classified frailty with only one tool, such as the Rockwood Clinical Frailty Scale (CFS) [36], electronic Frailty Index (eFI) [31]; frailty phenotype (FP) [37]; electronic Frailty Score (eFS) [5]; and an exception that utilized a combination of tools [21], which included FRS-26-ICD (frailty drawn from ICD-10 Clinical Modification), ECI (The Elixhauser Comorbidity Index (ECI), high-risk medications (10 risk classification, Beers Criteria, 2019), sociodemographic characteristics, healthcare, and insurance utilization. Another exception used a set of predictors variables, including clinical and socioeconomic aspects, and six target variables (mortality, disability, urgent hospitalization, fracture, preventable hospitalization, and accessing the emergency department with red code) [38].

ML algorithms have been used to predict frailty-derived indicators based on health-related data. The eFI, which is based on the deficit accumulation approach, was predicted using several ML algorithms such as DT, KNN, and SVM [31]. They analyzed the data of 592 patients and the best performance was obtained with SVM, the accuracy was 93.5%, sensitivity 97.8%, and specificity 89.1%. The SVM algorithm requires 70 input variables and they remarked that SVM may prove less feasible in clinical scenarios where rule-based models, such as DT models, may be more interpretable to clinicians but the results in terms of accuracy are the poorest (42.4%) with DT models.

Aponte-Hao, in 2021, proposed to use ML algorithms to predict the CFS score based on two-year electronic medical records (EMR). The CFS ranges from one to nine, with one having the label of “very fit” and nine labeled “terminally ill” (the highest degree of frailty); the frailty was predicted using a dichotomized indicator into frail or not frail with a cut-off of five from the original physician-rated CFS score. After the removal of features with low variance or high correlation, they reduced the total number of features from 5466 to 75. They used DT, LR, SVM, NB, NN, KNN, RF, and XGBoost models, and the XGBoost was the model with the best results of the eight models which were developed; it achieved the highest sensitivity (78.14%) and specificity (74.41%), but the F1-score was not shown when they used the best threshold that was achieved by using the most optimal thresholds determined using ROC curves [36].

An ML-based tool for stratification of FP based on one-year hospital discharge data was developed and validated (Pogam 2022). They created a clinical knowledge-driven eFS calculated as the number of deficient organs/systems among 18 critical ones identified from the ICD-10 diagnoses coded in the year before FP assessment. In addition, for eFS development and internal validation, they linked individual records of the cohort database to inpatient discharge data for an 11-year period. The best-performing model for predicting the dichotomized FP was the LR model with four predictors: age and sex at FP assessment, time since last discharge, and the eFS. The eFS score was associated with all adverse health outcomes of interest (death, prolonged length of hospital stay, number of hospitalizations, and nursing home admission within 12 months after FP assessment). They also conducted an external validation which confirmed that the eFS was a significant predictor of the 13 adverse outcomes [5].

Six frailty conditions (mortality, urgent hospitalization, disability, fracture, and emergency admission) were predicted with ML models (Tarekegn 2020). These models were assessed with a dataset that contains 1,095,612 subjects and 64 variables (58 input and 6 output variables). They resolved the imbalanced nature of the data through a resampling process and they performed a comparative study between the different ML algorithms: ANN, genetic programming (GP), SVM, DT, and RF. The obtained results show that the prediction performance of ML models significantly varies from problem to problem in terms of different evaluation metrics. The mortality prediction outcome showed higher performance with ANN (F1-score 0.79) and SVM (F1-score 0.78) than predicting the other outcomes. On average, over the six problems, the DT classifier showed the lowest accuracy, while other models (GP, LR, RF, ANN, and SVM) performed better. All models showed lower accuracy in predicting an event of an emergency admission with a red code than predicting fracture and disability. In predicting urgent hospitalization, only SVM achieved better performance (F1-score 0.76) [38].

ML models were also developed for predicting 30-day unplanned readmissions for elderly patients by integrating variables such as frailty and comorbidities (Mohanty 2022). The models were developed with data from 68,152 patients, consisting of 18,840 readmissions and 109,741 non-readmissions and containing 458 variables that were used for the prediction of readmission. The ML models compared were RF, XGBoost, CatBoost, and logistic regression, and a stacking classifier CatBoost outperformed the other models with an AUROC of 79% and F1 score of 71%. They performed an in-depth study of the model explainability by assessing the feature importance by means of the SHAP methods [21].

Moreover, a deep learning approach was followed to classify pre-frail/frail vs. non-frail older adults using heart rate response to physical activity [37]. They compared resting-state heart rate characteristics with heart rate monitoring without controlling for physical activities, the objective of the study. They assessed the performance of ML and deep learning models such as LSTM. The obtained results showed that LSTM outperformed other approaches. These results were obtained with a reduced sample size of 88 patients. This work shows that heart rate dynamics classification using LSTM deep learning models without any feature engineering may provide an accurate and objective marker for frailty screening [37].

## 4. Discussion

This paper presents a literature review of screening tools and clinical indicators for identifying early frailty and provides evidence for developing innovative tools through the focus on artificial intelligence. To our knowledge, this is the first narrative review summarizing and discussing frailty and ML for frailty screening. However, previous related research has been published on the relevance of the role in osteoporosis of AI models to model the risk of fragility fracture [32]. The selected studies were delivered between 2020 and 2022.

As mentioned earlier, the condition of frailty involves many domains that are not always easily identified, as well as the differences between them (e.g., cognitive, and physical domains). There are various instruments and identification criteria, thus hindering an accurate evaluation. Therefore, approaches that encompass all (or most) of these domains become relevant since they seem to have relevance in the early identification of the frail condition. Thus, ML is a promising approach, supported by recent studies.

The main findings of this study are that older age, females, clinical conditions (such as arthritis, hypertension, osteoporosis, and diabetes), high use of healthcare utilization, and adverse health outcomes (such as fractures, prolonged length of hospital stay, and number of hospitalizations) were the most significant predictive variables for the screening outcomes in frail persons. Previous studies reported that frailty was the most important predictor of rehospitalization and the second most important predictor of mortality in patients with cardiovascular disease [39]. Not surprisingly, the sociodemographic questions revealed importance. According to other studies, sociodemographic variables, namely, age and gender, are significant features [16,40]. Furthermore, in another recent study, age was the most important variable in predicting 90-day mortality and the second-most important variable for 30-day mortality [41].

The unsupervised learning methods are often used to process large databases, such as EMRs or large patient cohorts. Then, they can also cluster patients (subdividing them into groups) and characterize outliers or other essential features. Online electronic diagnosis systems are increasingly used by the population and healthcare professionals to a lesser extent. Most symptom checkers are ruled-based systems based on simple (conventional methods) decision trees. Therefore, ML is increasingly applied to EMRs in various health fields because they contain large, heterogeneous datasets that can be used to train disease detection or classification approaches using the supervised learning method [29].

Regarding algorithms, SVM was the most often applied ML method for frailty screening [5,31,36,38]. SVMs are competent in finding the best possible separation of different categories by familiarizing the weights of polynomial functions. ML models are typically trained using EMR or national cohorts. However, they require challenges to be applied effectively to information: the quantity and quality of the data. For example, deep neural networks commonly require massive training sets. Therefore, poor-quality training data (e.g., missing values) from EMRs will reduce the model’s overall quality [29]. Thus, ML has already shown clinically practical applications in frailty screening. It has the potential to support specialists in clinical and foster personalized health. Combined databases have the tremendous potential to provide sufficient data [29] because AI "feeds" on data. The more and better-quality data it accesses, the more it can excel at tasks. Some advanced algorithms need annotated data to ensure that those can learn. These annotated data depend on the health professionals. Relying on the algorithm utilized, they could require lots of annotated data. Thus, the dedicated contribution of “data annotators” is critical for the benefit of implementing AI in healthcare systems, as well as established standard methods to report data [28]. As is the case, Aponte Hao and colleagues include the RECORD Statement [42], promoting quality and transparency [36].

The accomplishment of AI and its place in clinical practice and healthcare depends on whether it can infiltrate the boundaries of an evidence-based approach, the lack of policies, and the lack of enthusiasm of health professionals to use it. On the other hand, the demand for AI to be implemented into everyday health professions is increasing among researchers, policymakers, clinical professionals, patients, hospitals, and developers. Therefore, it is essential to an integrated and appropriate multidisciplinary approach [28].

Some limitations regarding the data quality that impact the results should be noted. Although it is a validated tool for screening frailty, the CFS could not be advantageous to AI, because of the dichotomization, which fails to capture the severity of diseases, i.e., a person classified with pre-frail could be classified in the same category as a person with severe frailty. Therefore, an alternative could be to evaluate CFS as a continuous variable, bringing the distribution underlying the distribution of classes closer to the distribution of classes, then generating decision limits for the transformation back to the ordinal CFS to evaluate performance [36]. As well as in the CFS, the FRS-26 also be prone to bias in the quality of data and incapacitation of capturing the severity of symptoms [21]. In addition, the eFP, which could not classify frailty adequately, is often thought to reduce frailty to physical deficiencies and ignore mental and cognitive health problems [5]. In another study, they also merged pre-frail and fragile groups into a single group due to the limited number of fragile participants, and the size of the data (time series) was transformed to improve quality [37]. Moreover, other potential limitations include extensive missing data and test data being relatively small [31].

The accuracy of AI-generated results is highly dependent on the quality of the input data. Whether frailty is identified via the ML methods, very-high-quality data must be utilized if identification is ultimately proven accurate [31,41]. Further, efforts devoted to increasing the quality of the input data, such as standardized codes rather than free text and regular attention to data cleansing, may substantially improve the accuracy of the result obtained. The limited availability of high-quality data for training correctly labeled in medical claims, lack of detailed physiologic information, and indicators of the severity of comorbidities are inconsistently assigned, leading to a training set with underprivileged reproducibility and no “ground truth” to learn associations [28].

The heterogeneity of models makes it difficult to understand how accurate these methods might be in clinical practice or how reproducible they are in various clinical environments. The successful application of AI within the healthcare sphere does not remove the requirement for maintaining the quality of databases; instead, it is dependent on such activities. Another limitation is the studies using the codes ICD-10. The codes do not fully capture disease severity and might also miss out on essential elements of frailty such as weakness, polypharmacy, and need for support in everyday living. In addition, the potential variation in documentation and coding of diagnoses could contribute to measurement error (e.g., routine diagnosis and documentation of conditions such as delirium vary between clinicians and/or hospitals) [26].

In the future, with rapidly advanced wearables and monitoring technologies, we suggest researching other available frailty indicators—for example, using biosignals for postural control, gait assessment, and home-based frailty assessment. These types of data are also easily adapted to AI. They could be practical and feasible, such as falls prevention, an essential issue for frailty screening [15,33,43,44,45,46,47,48].

## 5. Conclusions

This review explores the tools and clinical indicators for frailty assessment and screening, through AI-based innovative tools. These existing tools, and clinical indicators for complex frailty, focusing on measures extracted from healthcare datasets were reviewed. The typical “health-professionally dependent” approaches for frailty screening could be adapted for technology-based approaches, such as eFI. The potential of AI techniques was explored; according to our findings, these methods can be used to identify risk factors to predict pre-frail or frailty. Thus, they facilitate the process to find the best treatment strategies for a person as well as frailty screening at the public health level. We suggest that databases collected from different populations be shared for improving the AI-based models.

This narrative review described the complex condition of frailty involving multi factor and summarized the indicators and the tools that were most used in the recent literature, as well as the AI models and the accuracies—making it easier for the developer and clinical to infer important data/variables for screening frailty. This review aims not to compare methods but to investigate the evidence for frailty screening. It was possible to conclude that the potential for ML to focus on frailty is immense, and offers an overabundance of new opportunities [29].

## Figures and Tables

**Table 1:** Selected studies using the machine learning methods.

First Author and Year	Sample Size and Age	Methods	Type of Data	Instrument (s)	Main Outcomes
Ambagtsheer 2020 [31]	592; ≥75	SVM; DT; KNN	Administrative records	Electronic Frailty Index	Arthritis; diabetes; hypertension; osteoporosis; vision issues; PAS score; Cornell scale; VBC; PBC; WC.
Aponte-Hao 2021 [36]	5466; ≥65	ENLR; SVM; KNN; NB; DT; RF; XGBoost; ANN	Electronic medical record	Rockwood Clinical Frailty Scale	Older; female; less likely to have no known CD.
Eskandari 2022 [37]	88; ≥65	LR; MLP; XGBoost; LSTM	Time-series ECG	Frailty Phenotype	HR dynamics.
Le Pogam 2022 [5]	469 int valid; 54,815 ext valid; 71.6 (mean)	BS-LR; Lasso-LR; RF; SVM	IR Lc65+ CHUV	Electronic frailty score	Older; female.
Mohanty 2022 [21]	76,000; ≥50	LR; RF; XGBoost; CatBoost; SC	electronic record data	Demo; FRS-26-ICD; ECI; H-RM; HIU	Prior readmissions; discharge to a rehabilitation facility; length of stay; comorbidities; frailty indicators (30-day readmission).
Tarekegn 2020 [38]	1,095,612; ≥65	ANN; GP; SVM; RF; LR; DT	administrative records	a set of variables (64)	Age (all problems); CI (mortality); number of urgent hospitalizations, femur and neck fracture (fracture problem); mental disease, poly-prescription and disease of the circulatory system (urgent hospitalization and preventable hospitalization); CI and number of urgent hospitalizations (emergency admission with red code).

Abbreviations: ANN: Artificial neural network; BS-LR: Best-Subsets; CatBoost: Category boost; CD: Chronic diseases; CI: Charlson Index; Demo: Demographic; DT: Decision tree; ECG: Electrocardiogram; ECI: The Elixhauser Comorbidity Index; EMR: Electronic medical records; ENLR: Elastic net logistic regression; Ext valid: External validation; FRS-26-ICD: The Frailty Risk Score 26 drawn from ICD-10 Clinical Modification (ICD-10-CM); ICD-10-CM: International Statistical Classification of Diseases and Related Problems 10th revision; GP: Genetic programming; HIU: Healthcare and insurance utilization; HR: Heart rate; HR-M: High-risk medications (Beers Criteria: 2019); German Modification; Int valid: Internal validation; IR Lc65+ CHUV: Individual Records Lc65+ cohort database to inpatient discharge data from Lausanne University Hospital (CHUV); KNN: K-nearest neighbors; Lasso-LR: Lasso-penalized logistic regression; LR: Logistic regression; LSTM: Long short-term memory; MLP: Multilayer perceptron; NB: Naive Bayes; PAS Score: Psychogeriatric Assessment Scales; PBC: Physical Behavior Checklist; RF: Random forest; SC: Stacking classifier; SVM: Support vector machine; VBC: Verbal Behavior Checklist; WC: Wandering checklist; XGBoost: Extreme gradient boosting.
